# Spontaneous Renal Pelvis Rupture: Unexpected Complication of Urolithiasis Expected to Passage with Observation Therapy

**DOI:** 10.1155/2013/932529

**Published:** 2013-10-01

**Authors:** Tuncay Tas, Basri Cakıroglu, Süleyman Hilmi Aksoy

**Affiliations:** ^1^Department of Urology, Star Medica Hospital, Orta Cami Mah. Cengiz Topel Meydanı No. 10, 5900 Tekirdag, Turkey; ^2^Department of Urology, Hisar Intercontinental Hospital, Saray Mahallesi Site Yolu Caddesi No. 7, Umraniye, 34768 Istanbul, Turkey; ^3^Department of Radiology, Hisar Intercontinental Hospital, Saray Mahallesi Site Yolu Caddesi No. 7, Umraniye, 34768 Istanbul, Turkey

## Abstract

Seventy percent of ureteral stones are located at distal ureter. Effective and safe passage of distal ureter stones is mediated by observation or medical expulsive treatment. Most of stones located at distal ureter pass spontaneously under observation; however, some are complicated with urinary tract infection, hydronephrosis, and renal function disturbances. Spontaneous perforation of the upper ureter is a rare condition that poses diagnostic and therapeutic problems. This case is reported, because the patient developed an unexpected spontaneous renal pelvis rupture (SRPR), while she was under observation and expected to pass her right ureteral stone spontaneously through hydration and analgesic treatment.

## 1. Introduction

Ureteral stones are 20% of urinary tract stones. Seventy percent of ureteral stones are located at distal ureter. Effective and safe passage of distal ureter stones is mediated by observation or medical expulsive treatment [1]. Most of stones located at distal ureter pass spontaneously under observation; however, some are complicated with urinary tract infection, hydronephrosis, and renal function disturbances. Urine extravasation from the renal collecting system or renal pelvis is a rare condition. When urine extravasation happens, it is generally related to obstruction, trauma, or previous urinary tract surgery [2]. This case is reported, because the patient developed an unexpected spontaneous renal pelvis rupture (SRPR), while she was under observation and expected to pass her right ureteral stone spontaneously through hydration and analgesic treatment.

## 2. Case Report

A 52-year-old woman has been admitted to the emergency department with acute flank pain for the last 6 days. Four days ago, the patient was followed in another clinic with observation option for spontaneous passage of distal ureteral stone. Hydration and twice daily diclofenac sodium 50 mg orally were recommended to the patient. The patient has been admitted to our emergency urology clinic with increased pain in the last 8 hours, increased nausea, and vomiting; no pathology was present in her medical history. 

**Figure 1:**
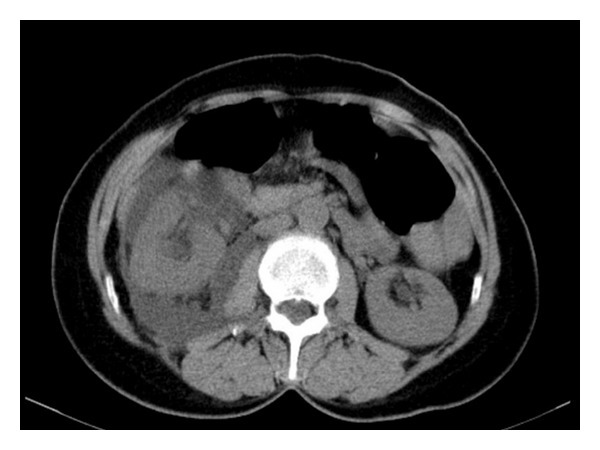
Free fluid around the kidney, especially near the pelvis renalis, is seen at CT scan without contrast-enhanced.

**Figure 2:**
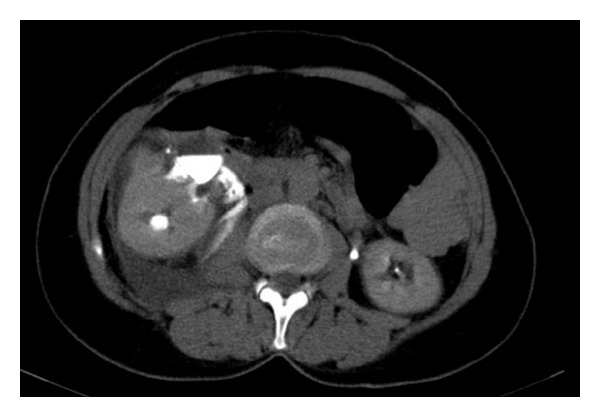
Free passage of contrast material from the pelvis renalis to around the kidney is seen at delayed phase of the contrast enhanced CT scan.

**Figure 3:**
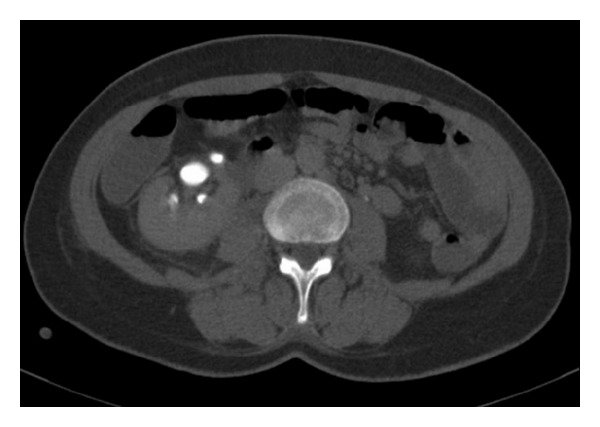
After treatment, no escape of contrast material from the pelvis renalis is seen at the delayed phase contrast enhanced control CT scan.

Physical examination revealed diffuse pain in the right abdomen with tenderness. Tenderness in costovertebral angle with local coolness in skin was present. In urinary ultrasonography, hydronephrosis and abundant fluid collection in perinephric area were seen. The left kidney was normal. In abdominal computerised tomography, rupture of renal pelvis in two points, peripheral fluid accumulation, contrast extravasation, and a 6 mm stone in right distal ureter were seen (Figures 1 and 2). In serum biochemical analysis hemoglobin level was 11.2 g/dL. Serum creatinine, urea, and other values were within the normal limits. After pain control, ureteroscopic lithotripsy, D-J stent replacement under scopy, and drain placement to perinephritic renal place were performed. Double-J ureteral stent was inserted under fluoroscopy, percutaneous drainage of the urinoma was performed, and 480 cc fluid was drained. In control CT of the patient, in the second postoperative day a marked improvement was seen (Figure 3). The patient was discharged after taking the drain. D-J stent was taken out a month later. Ultrasonography controls in outpatient clinic were normal.

## 3. Discussion

SRPR is an extremely rare condition. The most common abnormalities that have been reported are lithiasis, tumors, stricture, ruptured renal cysts, retroperitoneal fibrosis, congenital anomalies, postradiation scarring, pregnancy, renal transplants, vesicoureteral reflux, and urinary tract infection [2–4].

SRPR has the same symptoms as renal colic. The most common symptom in SRPR is renoureteral colic, flank pain, nausea, and vomitting. In physical examination, symptoms similar to abdominal pain pathogenesis, such as pyelonephritis, appendicitis, duodenal ulcer, biliary colic, and cholecystitis, may be seen.

Ultrasound and intravenous pyelogram are used in diagnosis and differential diagnosis. Intravenous contrast tomography is the most useful diagnostic tool. False negative results can be diminished by abdominal imaging.

Treatment is according to underlying pathology. Double J catheter or percutaneous nephrostomy is urinary diversion method to be used especially in the presence of small ruptures. Open surgery can be an option in difficult cases associated with extensive rupture of renal pelvis [5].

SRPR related to distal ureteral stone in a patient under observation was first noticed in our case which seems to be unique in the literature.

Ninety-five percent of 2–4 mm ureteral stones treated under observation treatment may pass spontaneously in 40 days. In cases of stones larger than 5 mm, 50% may need intervention [6]. In medical expulsive treatments in cases of distal ureteral stones with median sizes of 4.7–6.7 mm, 80% passage rate has been reported [7, 8]. Recommended period to wait for stones to pass under observation or medical expulsive treatment is 2 to 6 weeks [9]. More distally located stones, smaller and right side located stones, are more likely to pass spontaneously and require less intervention. Medical expulsive therapy is part of the established therapeutic means for ureteric calculi alongside observation, shock wave lithotripsy, ureteroscopy, and ureterolithotomy [10]. The best treatment choice in distal ureteral stones is still controversial. Choice depends on some factors including stone size, stone passage history, experience of the clinician, the patient's choice, available equipment, and cost.

In our case, the history of the patient showed no stone passage. Under the available factors, observation treatment was tested. In the 6th day pain which cannot be controlled with conservative treatment is seen.

In ureteral stones expected to pass under observation or treated with medical expulsive treatment, the possibility of renal pelvis rupture should be kept in mind. In sudden clinical deterioration, it can be the first thing to come to mind.
